# Single-Center 8-Years Clinical Follow-Up of Cladribine-Treated Patients From Phase 2 and 3 Trials

**DOI:** 10.3389/fneur.2020.00489

**Published:** 2020-06-17

**Authors:** Marcello Moccia, Roberta Lanzillo, Martina Petruzzo, Agostino Nozzolillo, Marcello De Angelis, Antonio Carotenuto, Raffaele Palladino, Vincenzo Brescia Morra

**Affiliations:** ^1^Department of Neuroscience, Reproductive Science and Odontostomatology, Multiple Sclerosis Clinical Care and Research Center, Federico II University, Naples, Italy; ^2^Department of Primary Care and Public Health, Imperial College, London, United Kingdom; ^3^Department of Public Health, Federico II University, Naples, Italy

**Keywords:** multiple sclerosis, cladribine, real-world, treatment, relapse, disability

## Abstract

**Background:** Cladribine is approved for the treatment of highly-active relapsing multiple sclerosis (MS), where it is also effective on disability progression. In the present single-center study, we aim to report on the 8-years clinical follow-up of 27 patients included in phase 2 and 3 clinical trials for cladribine.

**Methods:** We included patients exposed to cladribine (*n* = 13) or placebo (*n* = 14) in ONWARD, CLARITY, and ORACLE-MS trials, and followed-up at the same center after trial termination. Outcomes of long-term disease progression were recorded.

**Results:** During 8-year follow-up, patients treated with cladribine presented with reduced risk of EDSS progression (HR = 0.148; 95%CI = 0.031, 0.709; *p* = 0.017), of reaching EDSS 6.0 (HR = 0.115; 95%CI = 0.015, 0.872; *p* = 0.036), and of SP conversion (HR = 0.010; 95%CI = 0.001, 0.329; *p* = 0.010), when compared with placebo.

**Conclusions:** Our exploratory study provides additional evidence that cladribine may be useful to prevent or, at least, mitigate the risk of disability progression after 8 years.

## Background

Cladribine is a synthetic purine nucleoside analog, approved for the treatment of patients with highly active relapsing-remitting multiple sclerosis (RRMS), as defined by clinical and imaging features (1, 2). Short-course therapy with cladribine delays MS diagnosis after the first demyelinating event (3) and reduces relapse rate and inflammatory activity on MRI (2, 4).

Growing evidence supports cladribine could exert a neuroprotective effect, along with anti-inflammatory action. In clinical trials, cladribine treatment was associated with reduced brain atrophy and, accordingly, with reduced risk of disability progression over 2 years, compared with placebo (4, 5). More recently, in a propensity score–matched cohort, cladribine was more frequently associated with confirmed disability improvement after 1 year, compared with interferon-beta, fingolimod, and natalizumab (6). However, disability should be ideally assessed over a longer observation time (7–9), in particular for disease modifying treatments (DMTs) that produce durable effects on the immune system (e.g., cladribine) (1).

In the present single-center study, we aim to report on the 8-years clinical follow-up of 27 patients included in phase 2 and 3 trials for cladribine.

## Methods

### Study Design and Population

This is a retrospective analysis on prospectively collected data.

Inclusion criteria were (1) randomization in the CLARITY, OWARD, and ORACLE-MS trials in Federico II MS Center of Naples, Italy, for the entire study duration (i.e., 2-years treatment cycle); (2) follow-up visits at this center after trial termination.

All patients for this analysis (cladribine and placebo) were derived from the CLARITY, ONWARD, and ORACLE-MS trials. At the Federico II MS Center of Naples, we included 27 patients exposed to cladribine or placebo in ONWARD (*n* = 7), CLARITY (*n* = 10), and ORACLE-MS (*n* = 10) (Table 1). Allocation to active treatment or placebo was performed centrally by clinical trial CROs (Contract Research Organizations). Details on the trial design and results are fully reported elsewhere (3, 4, 10). Briefly, ONWARD and CLARITY are phase 2 and 3 clinical trials, respectively, including RRMS patients; ORACLE-MS is a phase 3 clinical trial including patients on their first clinical demyelinating event (either early RRMS or clinically isolated syndrome [CIS]). From retrospective review of medical records, all patients included in CLARITY, ONWARD, and ORACLE trials at our center had a diagnosis of RRMS according to 2010 criteria.

**Table 1:** Demographic and clinical features.

	**Placebo *(n* = 14)**	**Cladribine *(n* = 13)**	***p*-values**
Age*, years*	39.6 ± 10.9	39.3 ± 6.6	0.947
Sex*, female*	8 (57.1%)	9 (69.2%)	0.168
Disease duration^*^, years	8.9 ± 10.1	10.9 ± 6.6	0.545
Baseline EDSS	3.5 (1.5–6.0)	3.5 (1.5–5.5)	0.822
Baseline EDSS 0–2.0	4	2	
Baseline EDSS 2.5–4.0	4	6	
Baseline EDSS 4.5–6.0	6	5	
CLARITY/ONWARD/ORACLE-MS	5/3/6	5/4/4	0.776
Follow-up duration*, years*	7.1 ± 2.3	9.2 ± 1.8	0.015^*^
Post-trial DMT use (1st or 2nd line)	10 (71.4%)	9 (69.2%)	0.900
Post-trial 2nd line DMT use	3 (21.4%)	3 (23.1%)	0.918
**Post-trial clinical outcomes**			
Patients with relapse	5 (35.7%)	6 (46.1%)	0.404
Time to post-trial relapse*, years*	4.5 ± 2.9	5.4 ± 3.4	
Post-trial ARR	0.175 ± 0.323	0.167 ± 0.232	0.949
Patients with EDSS progression	8 (57.1%)	8 (61.5%)	0.017^*^
Time to EDSS progression*, years*	4.3 ± 2.2	7.2 ± 1.8	
Patients reaching EDSS 6.0	7 (50.0%)	5 (41.6%)	0.036^*^
Time to EDSS 6.0*, years*	5.2 ± 2.0	7.3 ± 2.0	
Patients converting to SP	7 (50%)	5 (41.6%)	0.010^*^
Time to SP conversion*, years*	4.7 ± 2.4	7.7 ± 1.8	

*Table shows mean and standard deviation (age, disease duration, follow-up duration, time to study outcomes), median and range (baseline EDSS), or number and percent (sex, EDSS subgroups, clinical trial, patients reaching study outcomes) of different study variables, as appropriate. P-values are reported from t-test and chi-square test for comparisons in demographics, baseline clinical features, and post-trial follow-up and DMT use; from Cox regression models for long-term clinical outcomes; and from Poisson regression model for ARR*.

* *Time from symptom onset to clinical trial inclusion*.

The “Federico II” ethical standards committee on human experimentation approved the study and written informed consent was obtained from all participants.

During the follow-up, all patients were assessed every 3 months, or on the occurrence of a clinical relapse, by one assessor (VBM), according to clinical practice; during the clinical trials, VBM was the principal investigator, and as such, was responsible for study conduction, including clinical assessments. Patients and assessor were blind to the use of cladribine during and after trial termination.

### Treatment Exposure

In the ONWARD trial, patients were randomized to either cladribine 3.5 mg/kg and Interferon-beta1a 44mcg TIW, or placebo and Interferon-beta1a 44mcg TIW over 96 weeks. In the CLARITY and ORACLE-MS trials, patients were randomized to cladribine 5.25 mg/kg, cladribine 3.5 mg/kg, or placebo over 96 weeks (3, 4, 10, 11). For the present study, we grouped (i) patients treated with cladribine 5.25 mg/kg, cladribine 3.5 mg/kg, or cladribine 3.5 mg/kg add-on to Interferon-beta1a 44 mcg in the cladribine group; (ii) and patients treated with placebo or placebo add-on to Interferon-beta1a 44mcg in the placebo group. We did not perform sub-analyses on different cladribine regimens (3.5 or 5.25 mg/kg) considering that they showed similar efficacy in clinical trials (4), and in light of sample size constraints. Allocation to active treatment or placebo was derived from unblinding procedures after trial termination or, when this was not available, on absolute lymphocyte count at 2 months after start of treatment (12). As from inclusion criteria, all patients received cladribine treatment (or placebo) on the first and on the second year.

After the 2-years trial duration, patients discontinued the cladribine (or placebo) and were treated in accordance with clinical practice. Of note, at the time of trial termination, efficacy results on cladribine were not available, and, thus, patients were treated based on clinical criteria (e.g., clinical and/or MRI activity) and/or patient preference (e.g., fear of returning to pre-clinical trial disease activity), under the supervision of the same treating physician (VBM).

### Clinical Outcomes

Age, sex, and disease duration (time from symptom onset to clinical trial inclusion) were recorded at baseline. During an average follow-up of 8.2 ± 2.3 (3.8–12.0) years, the patients were evaluated every 3 months, or at the occurrence of a clinical relapse, by an Expanded Disability Status Scale (EDSS) qualified neurologist blinded to the use of cladribine. The following major clinical outcomes were recorded during the follow-up period:

- Occurrence of clinical relapse, time to the first post-trial relapse, and annualized relapse rate (ARR); relapsing patients presented with a range of motor/sensory symptoms and met commonly used standards for relapse as determined by clinical neurologists in the clinical practice and trials (7);
- EDSS progression (1 point if baseline EDSS ≤ 5.5, or 0.5 point if ≥6.0, confirmed after 12 months, and independent of relapse), and time to EDSS progression;
- Reaching of EDSS 6.0 (confirmed after 12 months, and independent of relapse), and time to EDSS 6.0 (for long-term follow-up studies, reaching EDSS 6.0 is a milestone of disease progression, since patients do not improve after the said milestone is reached) (13);
- SP conversion (MS was considered as SP when a progressive accumulation of disability occurred after an initial relapsing course, and was associated with a worsening of the same functional system, independently from relapse activity [confirmed after 12 months]), and time to SP conversion (7–9).

### Statistical Analyses

Mean and standard deviation, median and range, and proportions were calculated for treatment groups. Preliminary comparisons between cladribine- and placebo-treated patients were performed with *t*-test and chi-square test, as appropriate. Cox regression models were employed to assess differences in rates of relapse occurrence (time to the first post-trial relapse); EDSS progression; reaching of EDSS 6.0; and SP conversion; results were reported as adjusted Hazard Ratios (HR) with 95% confidence interval (95%CI). For relapses, we run two additional Cox regression models including relapses from baseline to year 4 and, then, from year 4–year 8. Cox regression models automatically accounted for differences in follow-up duration, by measuring the effect on the time-at-risk, rather than the actual time-at-risk. A Poisson regression model was employed to evaluate the association between cladribine treatment and ARR; adjusted coefficients (Coeff) and 95%CI were subsequently calculated. Covariates included in the statistical models were age, sex, disease duration, baseline EDSS, relapses during trial duration, and clinical trial (CLARITY, ONWARD, and ORACLE).

A statistician was blinded to randomization codes. Results were considered statistically significant for *p* < 0.05. Stata 15.0 was used for data processing and analysis.

## Results

**Figure 1:**
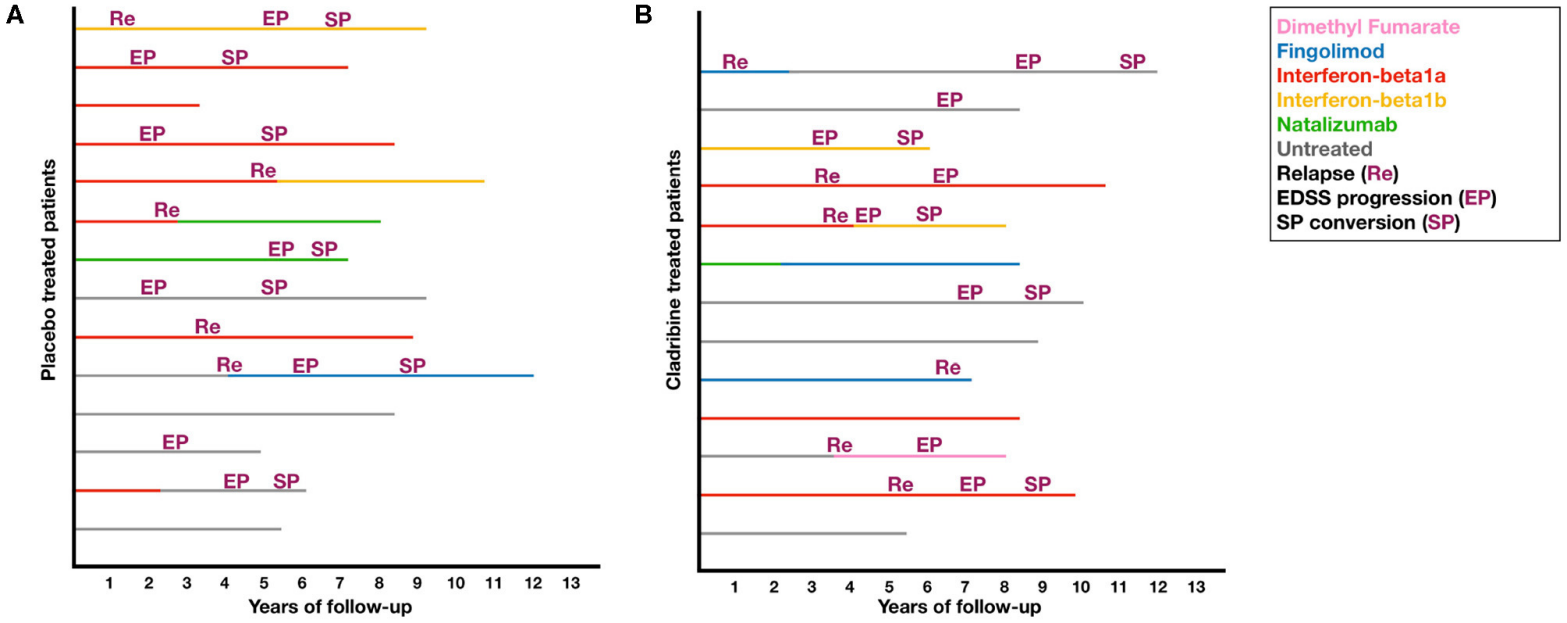
Post-trial treatments and study outcomes. Plots shows post-trial treatments and study outcomes in placebo **(A)** and cladribine-treated patients **(B)**.

Demographic and clinical features are reported in Table 1. At baseline visit, placebo- and cladribine-treated patients were similar in age, sex, disease duration, and EDSS and were equally distributed between trials. Patients were exposed to either placebo or cladribine during a similar time period (all patients received the 2-year cycle of cladribine). After trial completion, cladribine-treated patients were followed-up for a longer time, compared with placebo (over the follow-up period, one patient in the placebo group moved abroad, and no standardized clinical information is available). After trial completion, placebo- and cladribine-treated patients received similarly-active treatments; descriptive data on post-trial treatments and study outcomes are reported in Figure 1.

**Figure 2:**
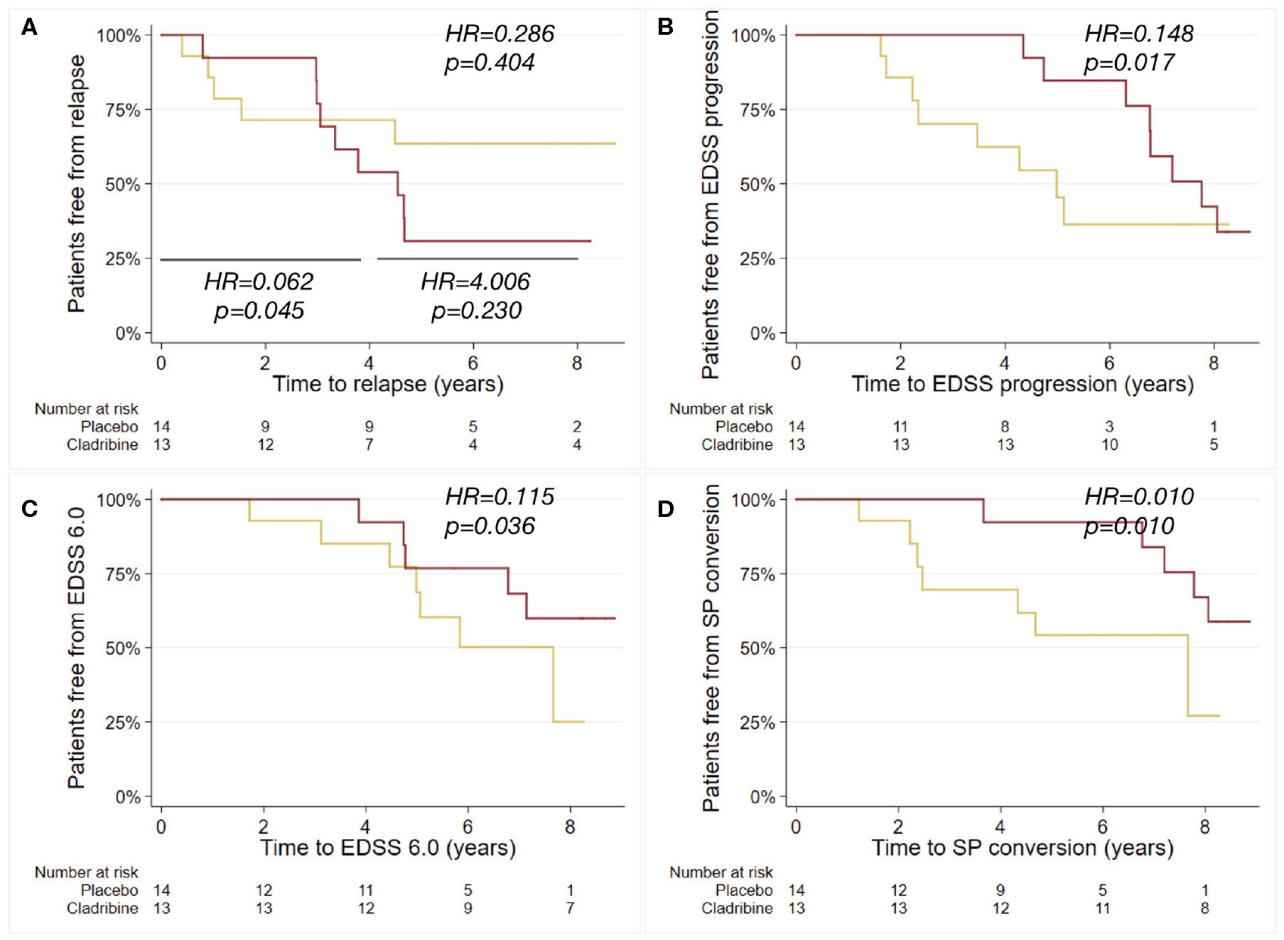
Kaplan-Meier curves for the probability of relapse, EDSS progression, reaching EDSS 6.0 and SP conversion. Kaplan-Meier plots estimating the probability of relapse after trial termination *(further sub-analyzed from baseline to year 4, and from year 4 to year 8)* **(A)**, of EDSS progression **(B)**, of reaching EDSS 6.0 **(C)**, and of SP conversion **(D)**, in relation to the exposure to cladribine (red) or placebo (yellow) during the 2-years trial duration. Hazard ratios (HR) and *p*-values are shown from Cox regression models.

Patients treated with cladribine presented with reduced risk of EDSS progression (HR = 0.148; 95%CI = 0.031, 0.709; *p* = 0.017), of reaching EDSS 6.0 (HR = 0.115; 95%CI = 0.015, 0.872; *p* = 0.036), and of SP conversion (HR = 0.010; 95%CI = 0.001, 0.329; *p* = 0.010), when compared with placebo (Table 1; Figure 2). No differences were found between placebo- and cladribine-treated patients in post-trial ARR (Coeff = 0.006; 95%CI = −0.217, 0.231; *p* = 0.949), and relapse risk (time to first post-trial relapse) (HR = 0.286; 95%CI = 0.415, 0.984; *p* = 0.404) (Table 1; Figure 2). When analyzing from baseline to year 4, patients treated with cladribine presented with reduced relapse risk, when compared with placebo (HR = 0.062; 95%CI = 0.004, 0.937; *p* = 0.045), whilst no differences were found from year 4 to year 8 (HR = 4.006; 95%CI = 0.415, 38.636; *p* = 0.230).

## Discussion

The present single-center study found reduced risk of 8-year disability progression and SP conversion in patients treated with cladribine during phase 2 and 3 trials, when compared with placebo.

A therapeutic lag on disability outcomes has been hypothesized for a number of treatments (14, 15) and might apply to cladribine as well. Cladribine induces a modest depletion of T-lymphocytes and a long-lasting depletion of B memory lymphocytes and subsequent humoral response (2, 16, 17), possibly persisting up to 10 years after treatment (as measured with intrathecal oligoclonal bands) (18). Not least, cladribine crosses the blood-brain barrier and reaches cerebrospinal fluid concentrations up to 25% plasma concentration or more, in case of blood-brain-barrier disruption (1, 2). As such, a more benign clinical progression could be the long-term consequence of modulation of the inflammatory response within the central nervous system (1, 2).

ONWARD, CLARITY, and ORACLE-MS clinical trials showed a significant difference in relapse risk between cladribine and placebo (3, 4, 11). In line with this, we showed reduced relapse risk within the first 4 years, but not over 8-years follow-up. This is possibly due to the use of other DMTs after cladribine discontinuation in our real-life cohort, in absence of knowledge on long-lasting effects of cladribine on reducing the frequency of relapses at the time of study conduction, and to the long-term follow-up, with patients being possibly out of anti-inflammatory effects of cladribine after 4 years (19). In particular, after trial termination, placebo- and cladribine-treated patients presented with similar treatment requirements (e.g., 70% were treated with an additional DMT, of which 20% with second line DMTs). In the ONWARD (11) and in the CLARITIY studies (4), cladribine also proved effective on disability progression. As such, our clinical observation provides additional evidence that cladribine may be useful to prevent or, at least, mitigate progressive disability accrual in the long term, and also in more advanced patients (e.g., in our cohort a number of patients presented with relatively-high baseline EDSS). Long-term reduced disability progression is in keep with cladribine effect on brain atrophy, as shown in the CLARITY study (5). Interestingly, our cohort presented with a dissociation between inflammatory activity (i.e., relapses, ARR) and disability outcomes (i.e., EDSS progression, EDSS 6.0, SP conversion), further suggesting that early demyelinating events are not necessarily related to the long-term disability progression (20–22).

Limitations of our study include sample size and the use of clinical trials with different inclusion criteria. However, this is an exploratory single-center experience, not aiming to provide final evidence on this topic. Also, due to sample size constraints, we did not perform sub-analyses on different cladribine regimens (3.5 vs. 5.25 mg/kg) and add-on treatments (Interferon-beta1a 44mcg vs. placebo), though possibly accounted for by the inclusion of clinical trial as a covariate in the statistical models. Similarly, we were not able to analyze statistically post-cladribine DMT sequencing, although we did not detect any obvious differences, at descriptive level, in the rate of patients who remained untreated, and who received more active DMTs (Table 1 and Figure 1). Unfortunately, MRI measurements were not collected in a standard fashion (e.g., different time intervals, field strengths, scans, acquisition protocols, etc.) and so were not included in the present study.

In conclusion, in this exploratory study, we provided preliminary evidence of cladribine efficacy on disability outcomes in the long-term, suggesting that cladribine might exert beneficial effects on neurodegenerative clinical aspects of MS. Present findings will require further validation on larger samples.

## Data Availability Statement

The datasets generated for this study are available on request to the corresponding author.

## Ethics Statement

The studies involving human participants were reviewed and approved by Federico II University Ethics Committee. The patients/participants provided their written informed consent to participate in this study.

## Author Contributions

MM, RP, and VM designed the study. RL, MP, AN, MD, and AC collected data. RP and VM analyzed data. MM, RL, MP, AN, MD, and AC drafted a preliminary version of this manuscript. All authors read and approved the final manuscript.

## Conflict of Interest

The authors declare that the research was conducted in the absence of any commercial or financial relationships that could be construed as a potential conflict of interest. The reviewer DP declared a past co-authorship with several of the authors, AC, MM, RL, and MP to the handling editor.
